# Maximal and sub-maximal exercise tests alter PBMC microRNA expression: insights into sport- and sex-specific variations

**DOI:** 10.3389/fphys.2025.1583870

**Published:** 2025-07-17

**Authors:** Guy Shalmon, Guy Shapira, Rawan Ibrahim, Ifat Israel-Elgali, Meitar Grad, Rani Shlayem, Ilan Youngster, Mickey Scheinowitz, Noam Shomron

**Affiliations:** 1 Sylvan Adams Sports Institute, School of Public Health, Gray Faculty of Medical and Health Sciences, Tel Aviv University, Tel Aviv, Israel; 2 Gray Faculty of Medical and Health Sciences, Tel Aviv University, Tel Aviv, Israel; 3 Edmond J Safra Center for Bioinformatics, Tel Aviv University, Tel Aviv, Israel; 4 Department of Biomedical Engineering, Faculty of Engineering, Tel Aviv University, Tel Aviv, Israel; 5 Pediatric Infectious Diseases Unit, The Center for Microbiome Research, Shamir Medical Center, Tel Aviv, Israel

**Keywords:** PBMC microRNA expression, peripheral blood mononuclear cells, endurance athletes, runners, cyclists, females

## Abstract

**Introduction:**

Studying microRNAs in the immune cells of athletes offers a novel perspective on the molecular regulation of immune function and recovery, potentially uncovering strategies to enhance performance and resilience to physical stress. However, PBMC microRNA expression in endurance athletes, such as runners and cyclists, remains underexplored, especially with regard to sex differences.

**Aims:**

(i) Assess sport- and sex-specific differences in PBMC microRNA expression induced by acute maximal and sub-maximal aerobic exercise in runners and cyclists and (ii) examine correlations between PBMC microRNAs and exercise performance. Methods: A total of 58 healthy athletes were included: 22 runners (9 females), 18 cyclists (9 females), and 18 active controls (9 females). Participants underwent VO2max and time-to-exhaustion tests, with blood samples collected pre- and post-exercise to analyze PBMC microRNA levels.

**Results:**

Runners exhibited a higher microRNA response than cyclists or controls, with significant sex-based differences. After VO2max test, 279 microRNAs (255 upregulated) were altered in runners, compared to only seven microRNAs (none upregulated) in cyclists. Exercise intensity and duration had sport-specific effects on microRNA expression. Time-to-exhaustion in runners and weekly training volume in both groups were significantly associated with changes in PBMC microRNA profiles.

**Conclusion:**

This study reveals that PBMC microRNA expression in response to maximal and sub-maximal exercise tests are sport- and sex-specific, providing new insights into the molecular adaptations of endurance athletes and their relationship to athletic performance.

## 1 Introduction

MicroRNAs (miRNAs) are small non-coding RNAs (18–24 nucleotides) that regulate gene expression by binding to messenger RNAs (mRNAs), promoting their degradation or inhibiting translation. This regulation is essential for cellular homeostasis, immune function, and tissue repair (Rocchi et al., 2021). Measuring microRNA expression before and after exercise offers valuable insights into athletes’ physiological adaptations (Da Silva et al., 2020). Exercise-induced changes in microRNA levels may reflect the body’s adaptive responses, and these alterations have been observed in various biological samples including serum, plasma, saliva, and muscle biopsies (Da Silva et al., 2020). However, studies specifically examining microRNAs in peripheral blood mononuclear cells (PBMCs) remain scarce (Radom-Aizik et al., 2012; Carbonare et al., 2022). PBMCs—comprising lymphocytes, monocytes, and dendritic cells—are critical immune cells that transiently increase in circulation following acute exercise. Endurance training may enhance the expression of specific PBMC microRNAs associated with immune function, recovery, and adaptation (Radom-Aizik et al., 2012; Carbonare et al., 2022). Yet, comparative studies of microRNA expression between different endurance sports or between sexes are still lacking.

Runners and cyclists display distinct biomechanical and physiological characteristics. Runners often exhibit higher VO_2_max on a treadmill, whereas cyclists may reach similar VO_2_max levels on a cycle ergometer (Millet et al., 2009). Sport-specific adaptations, such as differences in ventilatory thresholds, cardiac output, and muscle recruitment, can influence submaximal parameters even without changes in VO_2_max. Notably, running imposes impact forces absent in cycling (Millet et al., 2009), which may differentially affect physiological responses and microRNA expression.

Biological sex also shapes physiological traits through hormonal, chromosomal, and epigenetic interactions (Khramtsova et al., 2019; Oliva et al., 2020). Recently, microRNAs have emerged as important mediators of sex-specific biological differences (Cui et al., 2018), making it essential to explore microRNA expression in male and female athletes under exercise stress.

This study aimed to determine the PBMC microRNA expression profile in female and male runners and cyclists before and after (i) a maximal exercise (VO_2_max) test, and (ii) a submaximal exercise (85% of VO_2_max) until exhaustion.

## 2 Methods

### 2.1 Study design and participants

Each subject received information about the study and signed an informed consent form after approval from the Tel Aviv University Ethics Committee (approval No. 0003766-1). All informed consent forms signed by the subjects are in the files of the principal researcher at Tel Aviv University, Israel.

We enlisted endurance runners and cyclists from structured, non-professional competitive sports teams in Israel, which are registered with national athletic or cycling associations. These teams operate within organized training frameworks, led by certified coaches, and participate in regular weekly training sessions, as well as local and national competitions throughout the year. Although the athletes are not professional in the contractual or salaried sense, they train and compete at a high level of commitment and performance. To further support their classification as competitive endurance athletes, we used objective inclusion criteria: runners were required to run at least 50 km per week (mean 67 ± 15.6 km), and cyclists to cycle at least 120 km per week (mean 174 ± 54 km). According to self-reports, the athletes trained at an intensity of 80% or more of their maximum heart rate. The runners had a mean age of 43 ± 6.5 years and a body mass index (BMI) of 23.2 ± 2.61. The cyclists had a mean age of 46 ± 7 years and a BMI of 22.9 ± 3.25. The ethnic origin of all the participants in this study was Ashkenazi Jews (originating from Europe and North America).

The control group participants were selected from the general population and consisted of healthy, active individuals who engaged in light physical activity. Their weekly walking or running volume was less than 5 km per week, performed for recreational purposes to maintain general health and lifestyle rather than for competition. The control group had a mean age of 41 ± 7.4 years and a BMI of 23.9 ± 4.01.

To ensure comparability between athletes and the control group, participants were selected to match as closely as possible in terms of age, BMI, and health status, with the primary difference being the level of physical activity. Inclusion criteria were: (1) age between 30 and 55 years. Efforts were made to recruit participants of similar average age in all study groups, resulting in a comparable age distribution across the study arms; (2) BMI within the normal range (20.0–24.9 kg/m^2^); (3) omnivorous dietary pattern to reduce dietary variability; and (4) self-reported good health, with no history or current evidence of chronic diseases, including cardiovascular, metabolic, gastrointestinal, or autoimmune disorders.

Exclusion criteria included: (1) smoking or use of nicotine products; (2) use of medications or dietary supplements known to influence vascular function, immune response, or gut microbiota (e.g., antibiotics, probiotics, prebiotics, corticosteroids) in the 3 months before the study; (3) adherence to vegetarian, vegan, ketogenic, or other specialized diets; and (4) recent participation in another clinical or dietary intervention study.

An online survey was completed by each participant, which provided detailed information about their diet and training exercises. Participants were instructed not to consume any pre-workout supplements, such as caffeine or nitric oxide supplements, drink coffee, and avoid any ergogenic dietary supplements that could affect their vascular function and enhance athletic performance for 72 h before their arrival for the exercise tests. They were also required to fast for 12 h before the tests to ensure all participants arrived at a comparable energy status.

### 2.2 Exercise tests

#### 2.2.1 Maximal exercise test (VO2max test)

Each participant underwent a maximal exercise test to assess their aerobic capacity (“exercise test A”). Maximal oxygen uptake (VO_2_max) and ventilatory thresholds (VT1 and VT2) were determined using a metabolic cart (Quark CPET, COSMED S.r.l., Rome, Italy) (Evans et al., 2015; Shephard, 1984). The system was calibrated prior to each test session according to the manufacturer’s specifications, including gas and flowmeter calibration. Cyclists performed the test on a stationary SRM Ergometer (SRM GmbH, Jülich, Germany), starting at an initial workload of 80 W. The workload increased incrementally by 20 W every minute, while participants were instructed to maintain a pedaling cadence of 80 revolutions per minute (RPM). The test was terminated at volitional exhaustion or when cadence dropped below 60 RPM despite encouragement. Runners and control participants performed a treadmill running test using an h/p/cosmos Saturn 300 treadmill (h/p/cosmos sports and medical gmbh, Nussdorf-Traunstein, Germany). The test began at a speed corresponding to approximately 50% of the participant’s estimated running economy, increasing by 1 kph every minute until volitional exhaustion. This protocol was selected to reflect the primary mode of physical activity in these groups. Control participants performed the treadmill test rather than a cycling test, as their habitual activity involved walking or light running and not cycling. To standardize intensity across individuals, all tests were individualized and based on each participant’s fitness level, with intensity progression tailored to elicit maximal effort within 8–12 min of exercise.

#### 2.2.2 Submaximal exercise test

One week later, participants completed a submaximal constant-load exercise test (“exercise test B”) to evaluate time-to-exhaustion at 85% of their previously determined VO_2_max (Laursen et al., 2007). Runners and control subjects performed the test on the same h/p/cosmos treadmill used for the maximal test, while cyclists used the SRM Ergometer. In the present study, differences in microRNA expression were assessed as within-group percentage changes in response to exercise. Specifically, post-exercise microRNA expression levels were compared to pre-exercise levels within each group: runners were evaluated before and after running, and cyclists were evaluated before and after cycling. Each group was assessed in its respective sport-specific exercise modality. Heart rate (HR) was continuously monitored using a POLAR watch (Polar Electro Oy, Kempele, Finland), and capillary blood lactate concentrations were measured every 5 min during the test using a Lactate Scout + handheld analyzer (EKF Diagnostics GmbH, Barleben, Germany). The test was terminated at volitional exhaustion or upon any signs of physiological distress, in accordance with safety protocols.

### 2.3 Blood collection, PBMC isolation, and PBMC microRNA analysis

#### 2.3.1 Blood collection

Peripheral blood samples were collected from participants following standard venipuncture procedures before and immediately after each exercise test (A and B). Resting values served as baseline levels. Blood was drawn into EDTA-containing tubes (purple cap Vacutainer™ tubes, BD Biosciences, Franklin Lakes, NJ, United States).

#### 2.3.2 Complete blood counts and white blood cells differential

The differential count of white blood cells (WBC) was performed using an automated hematology analyzer equipped with VCS technology (Volume, Conductivity, Scatter). Whole blood was analyzed on a DxH 800 Hematology Analyzer (Beckman Coulter, B46899), a device designed to provide complete blood counts and perform leukocyte differential using advanced laser light scatter and electrical impedance methods. This technology allows for precise classification of leukocyte subpopulations, enabling rapid and reliable analysis. The analyzer provides high-throughput capability, ensuring accurate and reproducible results in clinical settings.

#### 2.3.3 Blood separation for PBMC microRNA analysis

Blood samples were separated using UNI-SEP Lymphocyte Separation Tubes (Novamed, Jerusalem, Israel). Whole blood was transferred to the UNI-SEP tubes and centrifuged for 15 min at 2,700 revolutions per minute (RPM; 1524xg) to separate plasma and PBMC from red blood cells. PBMC and plasma were transferred to a new 15 mL tube and centrifuged for 10 min at 1700 RPM (604xg). Plasma was collected, and PBMC was gently re-suspended in 0.5 mL Phosphate buffered saline (PBS) and transferred to two 1.5 mL Eppendorf tubes (Eppendorf, Hamburg, Germany). Tubes were centrifuged for 5 min at 1700 RPM. The supernatant was discarded, and tubes with PBMC were stored at - 80 C.

#### 2.3.4 RNA extraction

Samples underwent RNA purification: PBMC samples were lysed using TRIzol reagent (Thermo Fisher Scientific, Waltham, MA, United States), followed by RNA separation using chloroform (Bio-lab, Israel) and centrifugation at 20,227 xg, at 4°C, for 20 min and isopropanol (Bio-lab, Israel) precipitation, with centrifugation at 20,227 xg, at 4°C, for 15 min. RNA pellet was washed twice with 75% ethanol (Bio-lab, Israel) diluted in DEPC-treated water (Bet Haemek, Israel) and centrifuged at 20,227 xg, at 4°C, for 5 min. Finally, RNA was diluted in DEPC-treated water (Bet Haemek, Israel). The final RNA concentration and purity were measured using a NanoDrop ND-1000 spectrophotometer (NanoDrop Technologies, Wilmington, DE, United States).

#### 2.3.5 RNA sequencing

As the sequencing and initial bioinformatics pipeline were performed externally by Macrogen Inc. (Seoul, South Korea), specific internal laboratory procedures may not be fully disclosed. However, based on standard practices associated with the SMARTer smRNA-Seq Kit and Macrogen’s publicly documented protocols, the following procedures were applied:

Total RNA extracted from PBMCs was used for library preparation with the SMARTer smRNA-Seq Kit (Takara Bio, Kusatsu, Japan), following the manufacturer’s instructions. Sequencing was performed on an Illumina HiSeq 2,500 platform (Illumina, San Diego, CA, United States) in paired-end mode, targeting a sequencing depth of approximately 20 million reads per sample. Adapter trimming and quality filtering were conducted using Cutadapt, and high-quality reads were aligned to the human reference genome (GRCh38) using Bowtie. miRNA quantification was carried out with miRDeep2, enabling the identification of both known and novel miRNAs. Normalization of read counts was performed using the trimmed mean of M-values (TMM) method. Quality control included FastQC analysis, removal of low-complexity reads, and evaluation of mapping rates. Differential expression analysis was conducted using the edgeR package. Benjamini-Hochberg correction was applied to control the false discovery rate (FDR), and miRNAs with FDR < 0.05 and an absolute log2 fold change ≥1 were considered significantly differentially expressed.

#### 2.3.6 Statistical analysis and bioinformatics

Raw sequencing data was processed using the pipeline nf-core/smrnaseq v2.24, and all downstream analysis was performed by a snake-make workflow using R libraries (version 4.3.1; Linux). Raw reads were trimmed and filtered using fastp, then aligned against the human miRBase reference by Bowtie1 and quantified using SAMtools. The batch effect between the two sequencing runs was corrected using negative binomial regression under ComBat-seq. Normalization and differential expression analysis were performed using DESeq2 v1.40.2, with scaled numeric outcome variables (BMI, HR, etc.). Associations between microRNA expression and continuous physiological variables (e.g., training volume, time-to-exhaustion) were modeled within the DESeq2 framework. Adjusted log2FoldChange (aLFC) values were moderated using the empirical Bayes-based ash. Figures were generated using extended ggplot2 v3.4.4.

A comparison of PBMC count among the three groups (runners, cyclists, and controls) was performed utilizing One-Way ANOVA in IBM SPSS Statistics (version 29), supplemented by post hoc Bonferroni analysis.

The threshold for statistical significance in differential expression was set at FDR < 0.05. No additional filtering was applied based on fold change, as the paired study design provides sufficient sensitivity to detect even subtle differences in expression levels.

Background parameters were tested separately for potential confounding effects. None were identified as statistically confounding. While some variables (e.g., training volume) varied between groups by design, other cohort parameters—such as age—were tightly controlled and exhibited a narrow range across all participants.

## 3 Results

### 3.1 Subject’s characteristics

The participants in the study are similar in terms of age and BMI. The average age of the runners, cyclists, and controls is 43.3, 45, and 39.4 years, respectively, with corresponding BMI values of 23.2, 22.9, and 23.9. These similarities in age and BMI suggest that the groups are reasonably comparable, thus minimizing potential confounding effects related to these variables. Table 1 presents the subject’s characteristics.

**TABLE 1:** Characteristics of the participants.

	Runners (*n* = 22)	Cyclists (*n* = 18)	Controls (*n* = 18)
**Sex**			
Females	9 (40.9%)	9 (50%)	9 (50%)
Males	13 (59.1%)	9 (50%)	9 (50%)
**Age**			
Mean (SD)	43 (6.5)	46 (7)	41 (7.4)
**Body Mass Index (BMI)**			
Mean (SD)	23.2 (2.6)	22.9 (3.3)	23.9 (4)
**Weekly training volume (km)**			
Mean (SD)	67 (15.6)	174 (54)	5 (0)

### 3.2 Results of the maximal and submaximal exercise test

The exercise tests assessed several measures to reflect the participants’ aerobic fitness. As expected, the cyclists’ and runners’ cardiopulmonary exercise test results were higher than the controls. Table 2 presents the cardiopulmonary indices for the runners, cyclists, and controls, while Table 3 provides the indices for male and female cyclists and runners.

**TABLE 2:** Cardiopulmonary indices of the groups.

Cardiopulmonary indices	Runners (*n* = 22)	Cyclists (*n* = 18)	Controls (*n* = 18)
VT1 (mL/kg/min)	35.8 ± 4.4	31.3 ± 5.9	26.4 ± 3.9
VT2 (mL/kg/min)	43.2 ± 5.7	40.6 ± 9	31.7 ± 4.5
VO2max (mL/kg/min)	46 ± 6.7	44.6 ± 9.6	36.7 ± 5.4
Time-to-exhaustion (min)	15.4 ± 6.7	11.3 ± 3.9	7.4 ± 3.1
Lactate max (mmol/L)	8 ± 1.6	9.5 ± 2.6	7.2 ± 2.8

VT1 = first ventilatory threshold, also known as the aerobic threshold. VT2 = second ventilatory threshold, also known as the respiratory compensation threshold (RCT), and the onset of blood lactate accumulation (OBLA). VO2max = maximal consumption of oxygen.

**TABLE 3:** Cardiopulmonary indices of the female and male cyclists and runners.

Cardiopulmonary indices	Female runners (*n* = 9)	Male runners (*n* = 13)	Female cyclists (*n* = 9)	Male cyclists (*n* = 9)
VT1 (mL/kg/min)	34.2 ± 4.4	37 ± 4.4	31.2 ± 6	31.5 ± 6.2
VT2 (mL/kg/min)	40.5 ± 4.3	45 ± 5.9	39.4 ± 8	42 ± 10.3
VO2max (mL/kg/min)	43.9 ± 5.4	47.5 ± 7.4	42.4 ± 8.3	46.8 ± 10.7
Time-to-exhaustion (min)	15.2 ± 7.8	15.6 ± 6.2	10.4 ± 4.1	12.2 ± 3.7
Lactate max (mmol/L)	7.7 ± 1.5	8.3 ± 1.8	8.4 ± 3	10.5 ± 1.9

VT1 = first ventilatory threshold, also known as the aerobic threshold. VT2 = second ventilatory threshold, also known as the respiratory compensation threshold (RCT), and the onset of blood lactate accumulation (OBLA). VO2max = maximal consumption of oxygen.

### 3.3 PBMC levels across groups and the effect of acute aerobic exercise on these levels

The measurement of PBMC levels in blood before and after the VO2 exercise test revealed an increase in the number of cells following exercise. This result was consistent across all three groups in the study (runners, cyclists, and controls), with the difference in PBMC levels before and after aerobic exercise (Delta (Δ) PBMCs) being statistically significant in each group (p-adj < 0.01). This effect was observed in both types of exercise, the maximal and the submaximal test, indicating that exercise intensity or duration did not serve as limiting factors. However, when comparisons between the groups (runners, cyclists, and controls) were made, no significant differences were found between them. Table 4 presents the PBMC levels in blood before and after aerobic exercise and the delta (Δ) between the two measurements. The values are reported in units of k/µL (thousands of cells per microliter of blood).

**TABLE 4:** The Difference in PBMC levels between runners, cyclists, and controls following max and sub-max exercise tests.

A. Maximal test	Runners	Cyclists	Controls	
PBMCs (K/μL) Pre-test A	0.5 ± 2.2	0.6 ± 2	0.6 ± 2.6	
PBMCs (K/μL) Post-test A	1 ± 4.9	1 ± 4.3	1 ± 5.2	
Delta (Δ) PBMCs (K/μL)	0.9 ± 2.7 (*p-adj* < 0.01)	0.8 ± 2.2 (*p-adj* < 0.01)	1 ± 2.5 (*p-adj* < 0.01)	No significant differences were found between the groups
**B. Submaximal Test**				
PBMCs (K/μL) Pre-test B	0.5 ± 2.2	0.5 ± 2	0.6 ± 2.7	
PBMCs (K/μL) Post-test B	1.3 ± 5	1 ± 4.7	1 ± 5.2	
Delta (Δ) PBMCs (K/μL)	1 ± 2.9 (*p-adj* < 0.01)	1 ± 2.6 (*p-adj* < 0.01)	0.8 ± 2.5 (*p-adj* < 0.01)	No significant differences were found between the groups

### 3.4 Effect of acute aerobic exercise on PBMC microRNA differential expression

Both exercise tests, the maximal and submaximal tests, resulted in differential expression of PBMC microRNAs, but the expression patterns differed, with variations observed between runners and cyclists.

Measurement of PBMC microRNAs before and after the maximal test (VO2max) revealed that exercise altered the expression of 279 microRNAs (FDR < 0.05; 255 upregulated) among the runners, while among the cyclists, the expression of 7 microRNAs (FDR < 0.05; none upregulated) was altered. Similarly, measurement of PBMC microRNAs before and after the submaximal test showed that exercise changed the expression of 232 microRNAs (FDR < 0.05; 161 upregulated) among the runners, and 85 microRNAs (FDR < 0.05; 75 upregulated) among the cyclists. These results indicate that the runners consistently exhibited a more pronounced response in terms of microRNA expression following both types of exercise.

Sex differences were observed in the differential expression of PBMC microRNAs, with males consistently showing higher expression levels than females. This difference was only evident in cyclists during the submaximal test, where males exhibited higher differential expression than females, whereas no such difference was seen in the maximal test. Regarding the maximal test, measurement of PBMC microRNAs before and after exercise revealed that among male runners, exercise altered the expression of 236 microRNAs (FDR < 0.05; 266 upregulated), while only 30 microRNAs (FDR < 0.05; 25 upregulated) were altered in female runners. In contrast, no changes in microRNA expression were observed in male cyclists, while female cyclists showed alterations in 7 microRNAs (FDR < 0.05; none upregulated). Similarly, in the submaximal test, among male runners, exercise changed the expression of 617 microRNAs (FDR < 0.05; 322 upregulated), while no changes were observed in female runners. Among cyclists, male participants exhibited altered expression of 183 microRNAs (FDR < 0.05; 142 upregulated), whereas no changes were detected in the female cyclists (FDR < 0.05). These results suggest that male endurance athletes generally exhibited a more pronounced microRNA expression response to acute aerobic exercise compared to female endurance athletes. However, the maximal exercise test response in female cyclists was greater than in males.

To determine whether the intensity and duration of exercise differentially affect PBMC microRNA expression in runners and cyclists, we compared the results of the maximal exercise test and the submaximal test. We identified distinct patterns between the athlete groups. Runners exhibited a greater differential expression following the maximal test compared to the submaximal test, with 279 microRNAs (FDR < 0.05; 275 upregulated) and 232 microRNAs (FDR < 0.05; 161 upregulated) being expressed, respectively. In contrast, cyclists displayed a higher differential expression after the submaximal test than the maximal test, with 85 microRNAs (FDR < 0.05; 75 upregulated) and seven microRNAs (FDR < 0.05; none upregulated) expressed, respectively. Overall, acute maximal exercise had a more significant impact on microRNA expression in runners, while acute sub-maximal exercise-to-exhaustion had a more pronounced effect on microRNA expression in cyclists.

Table 5 presents statistically significant data (FDR < 0.05) on the maximal and submaximal exercise-induced differential expression of PBMC microRNAs in runners, cyclists, and controls, with a further breakdown by male and female participants within each group.

**TABLE 5:** Differential expression of PBMC microRNAs (FDR <0.05) induced by maximal (A), submaximal (B), or both exercise tests.

Group	Sex	Test	Total miRNAs	Upregulated	Downregulated
Runners	Males	B	617	322	295
		A	236	226	10
		A+ B	0	0	0
	Females	A	30	25	5
		B	0	0	0
		A+ B	0	0	0
	Both	A	279	255	24
		B	232	161	71
Cyclists	Males	A+ B	113	109	4
		A	0	0	0
		B	183	142	41
	Females	A	7	0	7
		B	0	0	0
		A+ B	0	0	0
	Both	A	7	0	7
		B	85	75	10
Controls	Males	A	338	266	72
		B	89	83	6
		A+ B	263	219	44
	Females	A	22	19	3
		B	4	0	4
		A+ B	0	0	0
	Both	A	22	19	3
		B	40	25	15
All Groups	Males	A	417	300	117
		B	913	464	449
		A+ B	424	307	117
	Females	A	0	0	0
		B	0	0	0
		A+ B	0	0	0

Note (Table 5): Some test conditions (e.g., A, B, or A + B in female cyclists and controls) resulted in no differentially expressed miRNAs (0/0/0). These entries are included in summary form to maintain clarity and consistency in presentation.

**FIGURE 1:**
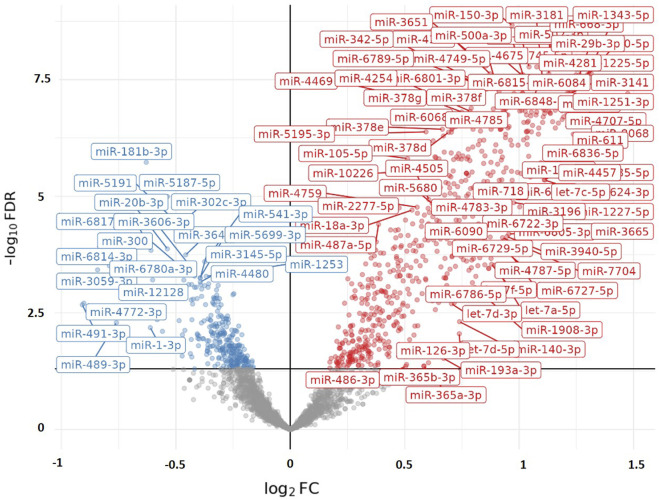
Volcano plot visualizing the differential expression of PBMC microRNAs (miRs) in response to Maximal (&/or Sub-Maximal exercise test(s) in runners compared to cyclists. The X-axis represents the adjusted log2Foldchange in microRNA expression between the two groups (runners vs. cyclists). A positive value of Log2FoldChange indicates an upregulation in microRNA expression (represented in red on the graph), while a negative value indicates a downregulation (represented in blue on the graph). The Y-axis represents the significance (−log10 [*FDR*]). The graph displays only microRNAs with statistical significance (*FDR* < 0.05).

**FIGURE 2:**
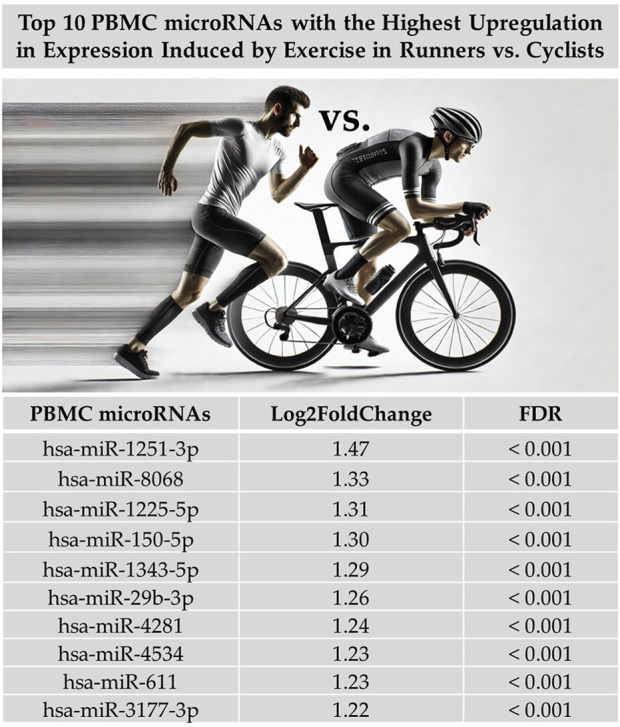
Top 10 PBMC microRNAs (miRs) with the highest upregulation in expression induced by maximal and submaximal exercise in runners compared to cyclists. Log2FoldChange measures the change in microRNA expression, where the fold change between the expression levels of a microRNA in two groups (runners vs cyclists) is calculated. The ‘Log2’ transformation is applied to convert the change into logarithmic base two units, making the data more suitable for comparison. Figure generated with graphical assistance from AI software (ChatGPT, version 4); no AI involvement in data analysis or interpretation.

When we examined the total effect of exercise on the differential expression of PBMC microRNAs by combining data from both exercise tests without separating them and disregarding differences in exercise intensity and duration, our analysis revealed that exercise-induced a greater differential expression in runners than cyclists. A total of 230 PBMC microRNAs were significantly altered by exercise in runners compared to cyclists (FDR < 0.05), with 176 showing upregulation and 54 showing downregulation. Figure 1 illustrates the differential expression of PBMC microRNAs in response to exercise in runners versus cyclists. Figure 2 presents the top 10 PBMC microRNAs with the highest upregulation in expression induced by exercise in runners compared to cyclists.

**FIGURE 3:**
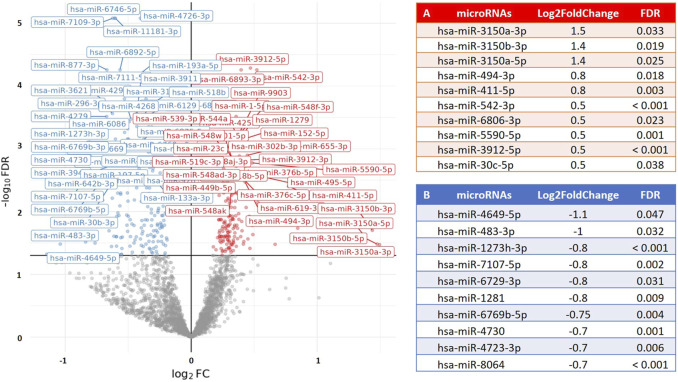
Volcano plot visualizing the association between time-to-exhaustion (min) and PBMC microRNA (miR) expression in runners. The results show differential expression of 195 PBMC microRNAs (FDR < 0.05; 75 upregulated) in relation to time-to-exhaustion. The X-axis represents the fold change in microRNA expression (Log_2_FoldChange), and the Y-axis represents the statistical significance (−log_10_ [FDR]). **(A)** The top 10 PBMC microRNAs with the highest upregulation in expression. **(B)** The top 10 PBMC microRNAs with the greatest downregulation in expression.

**FIGURE 4:**
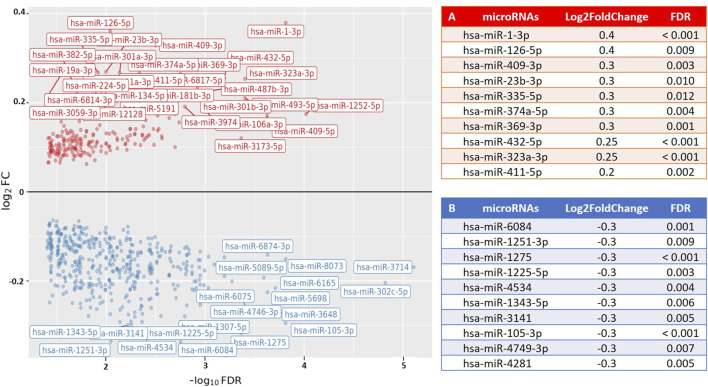
Volcano plot visualizing the association between weekly training volume (km) and PBMC microRNA (miR) expression in endurance athletes (runners and cyclists). The results show differential expression of 300 PBMC microRNAs (FDR < 0.05; 49 upregulated) in relation to weekly training volume. The X-axis represents the fold change in microRNA expression (Log_2_FoldChange), and the Y-axis represents the statistical significance (−log_10_ [FDR]). Results are valid for training distances of 50 km and above. **(A)** The top 10 PBMC microRNAs with the highest upregulation in expression. **(B)** The top 10 PBMC microRNAs with the greatest downregulation in expression.

To examine the relationship between post-exercise PBMC microRNA expression (following a time-to-exhaustion test) and exercise performance, we applied differential expression modeling using continuous covariates, including VO_2_max, blood lactate levels, and time-to-exhaustion. No significant associations were observed in any of the groups for VO_2_max or lactate levels. However, among runners, several microRNAs showed a significant association with time-to-exhaustion. Furthermore, when combining runners and cyclists into a single analysis, we identified a significant association between weekly training volume and PBMC microRNA expression, with 49 microRNAs found to be upregulated (Log_2_FoldChange = 0.2–0.38; FDR < 0.05). This suggests that increasing weekly training volume is accompanied by corresponding changes in PBMC microRNA expression, potentially reflecting physiological adaptations induced by training. Figure 3 illustrates the association between microRNA expression and time-to-exhaustion in runners, while Figure 4 displays the association between weekly training volume and microRNA expression across all endurance athletes.

## 4 Discussion

This study investigated sex- and sport-specific differences in PBMC microRNA expression among runners and cyclists in response to maximal and submaximal exercise. Our findings provide novel insights into the molecular mechanisms underlying endurance training adaptations, emphasizing the relevance of PBMC microRNAs in immune and physiological responses to exercise. PBMCs, integral to immune function, are affected by exercise-induced stressors, yet the expression profiles of PBMC-derived microRNAs in athletic populations remain underexplored.

PBMC counts increased significantly following both maximal and submaximal aerobic exercise in all groups, aligning with previous findings (Stampley et al., 2023). However, we observed no significant differences in PBMC counts between groups either at rest or post-exercise. In contrast, PBMC microRNA expression differed markedly, indicating that while cell counts rise similarly, the functional molecular response diverges based on sport and sex.

Strikingly, runners showed a much stronger PBMC microRNA response than cyclists—279 vs. 7 microRNAs after maximal exercise, and 232 vs 85 after submaximal exercise. This discrepancy likely reflects differences in biomechanical and physiological stressors, particularly eccentric muscle contractions and higher impact forces during running (Millet et al., 2009; Nieman et al., 2014), which can induce muscle damage, delayed onset muscle soreness (DOMS), and inflammatory responses (Nieman et al., 2014; Proske and Morgan, 2001; Peake et al., 2017). Most of the altered microRNAs in runners were upregulated, especially following maximal exercise (255/279), suggesting enhanced activation of regulatory networks associated with inflammation and mechanical stress adaptation.

Among the top 10 upregulated PBMC microRNAs in runners, two—miR-150-5p and miR-29b-3p—have recognized roles in immune modulation and exercise adaptation. miR-150 regulates B cell function and metabolism (Hu et al., 2023) and is upregulated following exercise (de Gonzalo-Calvo et al., 2015). It also correlates with VO_2_max and mitochondrial capacity in skeletal muscle (Dahlmans et al., 2017). Mechanistically, miR-150-5p serves as a key mediator of cardiovascular adaptations to moderate-intensity exercise, with circulating levels reflecting adaptive responses (Li et al., 2021). Exercise-induced shifts in microRNA profiles are recognized regulators of adaptation via mRNA modulation (Camera et al., 2016), positioning miR-150-5p as a candidate biomarker for training efficacy. In the absence of miR-150, CD8^+^ T cells fail to fully develop or differentiate into effectors; even memory-phase cells show impaired responses to secondary infection (Smith et al., 2015). In CD4^+^ T cells, the downregulation of antigen-specific miR-150 enhances survival, underscoring its role in T cell homeostasis and activation (Menoret et al., 2023). Similarly, miR-29b-3p, linked to tumor suppression and myogenesis, is modulated by exercise (Gazova et al., 2019; Afzal et al., 2024), suggesting a role in reduced cancer risk and tissue regeneration. The miR-29 family, including miR-29b-3p, exhibits tissue-specific responses to metabolic stress, with elevated expression associated with improved insulin sensitivity in some contexts (Dalgaard et al., 2022). These findings suggest that the upregulation of these microRNAs may reflect immune and mitochondrial adaptations triggered by intense mechanical loading in runners. The other eight microRNAs (e.g., miR-1251-3p, miR-8068) have been primarily studied in cancer contexts (Urabe et al., 2022; Li et al., 2020; Shao et al., 2019; Hamam et al., 2016) but may also play roles in immune stress responses (Borgan et al., 2011; Nie et al., 2020). Their exercise-induced expression in our study raises the possibility of broader roles in systemic adaptation to endurance training. While the current literature offers limited insight into the physiological functions of the remaining eight upregulated microRNAs, their consistent expression changes in response to acute endurance exercise in our study suggest that they may represent novel or underexplored components of the systemic stress response. The fact that these microRNAs—previously characterized mainly in pathological or oncological contexts—are differentially expressed in a physiological setting such as exercise raises important questions about their potential regulatory roles beyond disease. Further investigation is needed to determine whether these microRNAs participate in immune modulation, cellular repair, or metabolic adaptation pathways specific to endurance training. Thus, their identification in this context provides a foundation for future studies aimed at expanding the functional landscape of microRNA involvement in exercise biology.

It is worth noting that studies have demonstrated an association between physical activity and inflammatory markers linked to a reduced incidence of cancer (Mancini et al., 2024). Based on this body of evidence, we hypothesize that the expression of the top 10 PBMC-derived microRNAs identified in this study in athletes may indicate a potential link between physical activity, immune system adaptations, and a decreased risk of cancer-related morbidity. The fact that these microRNAs—previously characterized mainly in pathological or oncological contexts—are differentially expressed in a physiological setting such as exercise raises important questions about their regulatory functions beyond disease. These findings suggest that such microRNAs may play roles in immunometabolic regulation triggered by endurance exercise. Further studies are warranted to elucidate their specific contributions to the physiological adaptations associated with sustained aerobic training.

Two physiological mechanisms may underlie the differences between runners and cyclists: (1) biomechanical stress responses associated with eccentric versus concentric muscle contractions (Proske and Morgan, 2001). Running involves repeated eccentric loading, particularly during the landing phase, which induces greater mechanical strain on muscle fibers and connective tissues. This type of contraction is known to provoke more pronounced inflammatory and regenerative signaling cascades, potentially influencing the expression of circulating microRNA to a greater extent. (2) Divergent hemodynamic and cardiovascular demands (Millet et al., 2009). Compared to cycling, running imposes greater systemic physiological stress, including higher impact forces, increased cardiac output, and more comprehensive engagement of both lower and upper body musculature. These factors may result in broader systemic strain and, thus, a more complex transcriptional and post-transcriptional response, reflected in the differential regulation of circulating miRNAs.

We also identified clear sex-specific differences in PBMC microRNA responses. Male runners exhibited a stronger response than females (236 vs 30 microRNAs in maximal, 617 vs. 0 in submaximal testing), possibly due to hormonal influences on inflammation and muscle repair (Aragon-Vela et al., 2021). In contrast, female cyclists showed modest changes while males showed none, suggesting modality-specific interactions between sex and molecular response.

Interestingly, exercise intensity affected PBMC microRNA expression differently between sports. Runners showed a stronger response to maximal effort (279 vs. 232 microRNAs), while cyclists responded more to submaximal exertion (85 vs. 7), highlighting distinct adaptation profiles likely shaped by training specificity (Wahl et al., 2022).

Although most groups showed no significant association between PBMC microRNA expression and performance metrics (VO_2_max, lactate, time-to-exhaustion), runners displayed a specific association between time-to-exhaustion and several microRNAs. Additionally, weekly training volume was found to be associated with the differential expression of 49 PBMC microRNAs across all athletes. Among these, several microRNAs are known to be biologically relevant in the context of exercise physiology. For example, miR-1-3p and miR-126-5p, which were among the upregulated microRNAs, have been previously linked to oxidative stress, tissue repair, and angiogenesis (Eyileten et al., 2022; Ryningen et al., 2024), supporting their potential role in endurance adaptation.

In summary, this study demonstrated that (1) PBMC microRNA expression differs by sex and sport, with runners—especially males—showing the strongest response; (2) exercise intensity and modality distinctly shape molecular profiles; (3) key microRNAs, notably miR-150-5p and miR-29b-3p, may mediate immune and mitochondrial adaptations; and (4) training volume and performance outcomes are linked to specific microRNA responses. These findings underscore the potential of PBMC microRNA profiling to inform individualized training and recovery strategies in endurance athletes.

## 5 Limitations

Despite the comprehensive design of this study, several limitations must be acknowledged. First, the specific exercise protocols, though rigorously designed, represent acute exercise scenarios. While this effect is inherently interesting, it may vary over time as part of the physiological adaptations following physical exertion. It may not fully capture the complex microRNA dynamics associated with chronic, long-term endurance training. Therefore, it cannot necessarily be inferred as a chronic effect over time. To determine whether the observed differences between the groups are sustained, these measurements should be repeated multiple times over an extended period.

Second, the research was conducted with competitive endurance athletes from a specific geographical region (Israel) and specific ethnic origin (Ashkenazi Jews originating from Europe and North America). As the study sample was ethnically homogeneous, consisting solely of Ashkenazi Jewish participants from Israel, caution should be exercised when generalizing the findings to populations with different ethnic or cultural backgrounds. This might restrict the broader applicability of the results to diverse athletic populations.

Third, while the study controlled for factors such as pre-test fasting and supplement abstinence, individual metabolic variations, genetic predispositions, and potential unaccounted lifestyle factors could influence microRNA expression.

Fourth, although the total sample size was adequate for detecting general group differences, the number of participants in each specific subgroup (e.g., female runners and male cyclists) was relatively small. This may have limited the statistical power to detect more nuanced interaction effects within or between sex and sport type. Consequently, some potentially meaningful differences might not have reached statistical significance. Future studies with larger and more diverse cohorts are warranted to validate and expand upon these findings.

## 6 Conclusion

This study provides novel insights into the relationship between endurance exercise, PBMC microRNA expression, and athletic performance, suggesting that acute aerobic exercise-induced PBMC microRNA expression is probably sport- and sex-specific in runners and cyclists. Additional studies are required to understand the effect of chronic aerobic exercise on PBMC microRNA expression among these athletes.

## Data Availability

The datasets presented in this study can be found in online repositories. The names of the repository/repositories and accession number(s) can be found below: https://www.ncbi.nlm.nih.gov/geo/query/acc.cgi?acc=GSE288760. Accession number: GSE288760.

## References

[B1] Afzal M. Greco F. Quinzi F. Scionti F. Maurotti S. Montalcini T. (2024). The effect of physical activity/exercise on miRNA expression and function in non-communicable diseases-a systematic review. Int. J. Mol. Sci. 25 (13), 6813. 10.3390/ijms25136813 38999923 PMC11240922

[B2] Aragon-Vela J. Fontana L. Casuso R. A. Plaza-Diaz J. R H. J. (2021). Differential inflammatory response of men and women subjected to an acute resistance exercise. Biomed. J. 44 (3), 338–345. 10.1016/j.bj.2020.02.005 34140269 PMC8358195

[B3] Borgan E. Navon R. Vollan H. K. Schlichting E. Sauer T. Yakhini Z. (2011). Ischemia caused by time to freezing induces systematic microRNA and mRNA responses in cancer tissue. Mol. Oncol. 5 (6), 564–576. 10.1016/j.molonc.2011.08.004 21917534 PMC5528325

[B4] Camera D. M. Ong J. N. Coffey V. G. Hawley J. A. (2016)). Selective modulation of MicroRNA expression with protein ingestion following concurrent resistance and endurance exercise in human skeletal muscle. Front. Physiol. 7, 87. 10.3389/fphys.2016.00087 27014087 PMC4779983

[B5] Carbonare L. D. Dorelli G. Vigni V. L. Minoia A. Bertacco J. Cheri S. (2022). Physical activity modulates miRNAs levels and enhances MYOD expression in myoblasts. Stem Cell. Rev. Rep. 18, 1865–1874. 10.1007/s12015-022-10361-9 35316486 PMC9209351

[B6] Cui C. Yang W. Shi J. Zhou Y. Yang J. Cui Q. (2018). Identification and analysis of human sex-biased MicroRNAs. Genomics. Proteom. Bioinforma. 16 (3), 200–211. 10.1016/j.gpb.2018.03.004 30005964 PMC6076379

[B7] Dahlmans D. Houzelle A. Andreux P. Jorgensen J. A. Wang X. de Windt L. J. (2017). An unbiased silencing screen in muscle cells identifies miR-320a, miR-150, miR-196b, and miR-34c as regulators of skeletal muscle mitochondrial metabolism. Mol. Metab. 6 (11), 1429–1442. 10.1016/j.molmet.2017.08.007 29107290 PMC5681243

[B8] Dalgaard L. T. Sorensen A. E. Hardikar A. A. Joglekar M. V. (2022)). The microRNA-29 family: role in metabolism and metabolic disease. Am. J. Physiol. Cell. Physiol. 323 (2), C367–C377. 10.1152/ajpcell.00051.2022 35704699

[B9] Da Silva F. C. Da R. I. Andrade A. Costa V. P. Gutierres-Filho P. J. da Silva R. (2020). Effects of physical exercise on the expression of MicroRNAs: a systematic review. J. Strength Cond. Res. 34 (1), 270–280. 10.1519/JSC.0000000000003103 31877120

[B10] de Gonzalo-Calvo D. Davalos A. Montero A. Garcia-Gonzalez A. Tyshkovska I. Gonzalez-Medina A. (2015). Circulating inflammatory miRNA signature in response to different doses of aerobic exercise. J. Appl. Physiol. (1985) 119 (2), 124–134. 10.1152/japplphysiol.00077.2015 25997943

[B11] Evans H. J. Ferrar K. E. Smith A. E. Parfitt G. Eston R. G. (2015). A systematic review of methods to predict maximal oxygen uptake from submaximal, open circuit spirometry in healthy adults. J. Sci. Med. Sport 18, 183–188. 10.1016/j.jsams.2014.03.006 24721146

[B12] Eyileten C. Wicik Z. Fitas A. Marszalek M. Simon J. E. De Rosa S. (2022). Altered circulating MicroRNA profiles after endurance training: a cohort study of ultramarathon runners. Front. Physiol. 25 (12), 792931. 10.3389/fphys.2021.792931 35145424 PMC8824535

[B13] Gazova A. Samakova A. Laczo E. Hamar D. Polakovicova M. Jurikova M. (2019). Clinical utility of miRNA-1, miRNA-29g and miRNA-133s plasma levels in prostate cancer patients with high-intensity training after androgen-deprivation therapy. Physiol. Res. 68 (Suppl 2), S139–147. 10.33549/physiolres.934298 31842577

[B14] Hamam R. Ali A. M. Alsaleh K. A. Kassem M. Alfayez M. Aldahmash A. (2016). microRNA expression profiling on individual breast cancer patients identifies novel panel of circulating microRNA for early detection. Sci. Rep. 6, 25997. 10.1038/srep25997 27180809 PMC4867432

[B15] Hu Y. Z. Li Q. Wang P. F. Li X. P. Hu Z. L. (2023). Multiple functions and regulatory network of miR-150 in B lymphocyte-related diseases. Front. Oncol. 27 (13), 1140813. 10.3389/fonc.2023.1140813 37182123 PMC10172652

[B16] Khramtsova E. A. Davis L. K. Stranger B. E. (2019). The role of sex in the genomics of human complex traits. Nat. Rev. Genet. 20 (3), 173–190. 10.1038/s41576-018-0083-1 30581192

[B17] Laursen P. B. Francis G. T. Abbiss C. R. Newton M. J. Nosaka K. (2007). Reliability of time-to-exhaustion versus time-trial running tests in runners. Med. Sci. Sports Exerc 39, 1374–1379. 10.1249/mss.0b013e31806010f5 17762371

[B18] Li B. Zhang F. Li H. (2020). miR-1225-5p inhibits non-small cell lung cancer cell proliferation, migration and invasion, and May be a prognostic biomarker. Exp. Ther. Med. 20 (6), 172. 10.3892/etm.2020.9302 33101465 PMC7579767

[B19] Li D. Wang P. Wei W. Wang C. Zhong Y. Lv L. (2021)). Serum MicroRNA expression patterns in subjects after the 5-km exercise are strongly associated with cardiovascular adaptation. Front. Physiol. 12, 755656. 10.3389/fphys.2021.755656 34912238 PMC8667031

[B20] Mancini A. Orlandella F. M. Vitucci D. Luciano N. Alfieri A. Orrù S. (2024). Exercise’s impact on lung cancer molecular mechanisms: a current overview. Front. Oncol. 14, 1479454. 10.3389/fonc.2024.1479454 39555455 PMC11563951

[B21] Menoret A. Agliano F. Karginov T. A. Karlinsey K. S. Zhou B. Vella A. T. (2023)). Antigen-specific downregulation of miR-150 in CD4 T cells promotes cell survival. Front. Immunol. 14, 1102403. 10.3389/fimmu.2023.1102403 36817480 PMC9936563

[B22] Millet G. P. Vleck V. E. Bentley D. J. (2009). Physiological differences between cycling and running: lessons from triathletes. Sports Med. 39 (3), 179–206. 10.2165/00007256-200939030-00002 19290675

[B23] Nie X. He M. Wang J. Chen P. Wang F. Lai J. (2020). Circulating miR-4763-3p is a novel potential biomarker candidate for human adult fulminant myocarditis. Mol. Ther. Methods Clin. Dev. 12 (17), 1079–1087. 10.1016/j.omtm.2020.05.005 32478123 PMC7248292

[B24] Nieman D. C. Luo B. Dreau D. Henson D. A. Shanely R. A. Dew D. (2014). Immune and inflammation responses to a 3-day period of intensified running versus cycling. Brain Behav. Immun. 39, 180–185. 10.1016/j.bbi.2013.09.004 24055861

[B25] Oliva M. Munoz-Aguirre M. Kim-Hellmuth S. Wucher V. Gewirtz A. D. H. Cotter D. J. (2020). The impact of sex on gene expression across human tissues. Science 11 (6509), 369. 10.1126/science.aba3066 32913072 PMC8136152

[B26] Peake J. M. Neubauer O. Della Gatta P. A. Nosaka K. (2017). Muscle damage and inflammation during recovery from exercise. J. Appl. Physiol. (1985) 122 (3), 559–570. 10.1152/japplphysiol.00971.2016 28035017

[B27] Proske U. Morgan D. L. (2001). Muscle damage from eccentric exercise: mechanism, mechanical signs, adaptation and clinical applications. J. Physiol. 1, 333–345. 10.1111/j.1469-7793.2001.00333.x 11731568 PMC2278966

[B28] Radom-Aizik S. Zaldivar F. Leu S. Y. Adams G. R. Oliver S. Cooper D. M. (2012). Effects of exercise on microRNA expression in young males’ peripheral blood mononuclear cells. Clin. Transl. Sci. 5 (1), 32–38. 10.1111/j.1752-8062.2011.00384.x 22376254 PMC4664183

[B29] Rocchi A. Chiti E. Maiese A. Turillazzi E. Spinetti I. (2021). MicroRNAs: an update of applications in forensic science. Diagnostics 11, 32. 10.3390/diagnostics11010032 33375374 PMC7823886

[B30] Ryningen A. Rostad K. Ersvær E. Sjoholt G. Paulsen G. Gundersen H. (2024). Acute response in circulating microRNAs following a single bout of short-sprint and heavy strength training in well-trained cyclists. Front. Physiol. 15, 1365357. 10.3389/fphys.2024.1365357 38532845 PMC10963392

[B31] Shao Y. Liu X. Meng J. Zhang X. Ma Z. Yang G. (2019). MicroRNA-1251-5p promotes carcinogenesis and autophagy via targeting the tumor suppressor TBCC in ovarian cancer cells. Mol. Ther. 27 (9), 1653–1664. 10.1016/j.ymthe.2019.06.005 31278033 PMC6731176

[B32] Shephard R. J. (1984). Tests of maximum oxygen intake. A critical review. Sports Med. 1, 99–124. 10.2165/00007256-198401020-00002 6385195

[B33] Smith N. L. Wissink E. M. Grimson A. Rudd B. D. (2015)). miR-150 regulates differentiation and cytolytic effector function in CD8+ T cells. Sci. Rep. 5, 16399. 10.1038/srep16399 26549197 PMC4637875

[B34] Stampley J. E. Cho E. Wang H. Theall B. Johannsen N. M. Spielmann G. (2023). Impact of maximal exercise on immune cell mobilization and bioenergetics. Physiol. Rep. 11 (11), 15753. 10.14814/phy2.15753 37312242 PMC10264558

[B35] Urabe F. Matsuzaki J. Takeshita F. Kishida T. Ochiya T. Hirai K. (2022). Independent verification of circulating miRNA as diagnostic biomarkers for urothelial carcinoma. Cancer Sci. 113 (10), 3510–3517. 10.1111/cas.15496 35848873 PMC9530882

[B36] Wahl P. Bloch W. Proschinger S. (2022). The molecular signature of high-intensity training in the human body. Int. J. Sports Med. 43 (3), 195–205. 10.1055/a-1551-9294 34265857 PMC8885329

