# Correction: Maximal and sub-maximal exercise tests alter PBMC microRNA expression: insights into sport- and sex-specific variations

**DOI:** 10.3389/fphys.2025.1674758

**Published:** 2025-11-07

**Authors:** Guy Shalmon, Guy Shapira, Rawan Ibrahim, Ifat Israel-Elgali, Meitar Grad, Rani Shlayem, Ilan Youngster, Mickey Scheinowitz, Noam Shomron

**Affiliations:** 1 Sylvan Adams Sports Institute, School of Public Health, Gray Faculty of Medical and Health Sciences, Tel Aviv University, Tel Aviv, Israel; 2 Gray Faculty of Medical and Health Sciences, Tel Aviv University, Tel Aviv, Israel; 3 Edmond J Safra Center for Bioinformatics, Tel Aviv University, Tel Aviv, Israel; 4 Department of Biomedical Engineering, Faculty of Engineering, Tel Aviv University, Tel Aviv, Israel; 5 Pediatric Infectious Diseases Unit, The Center for Microbiome Research, Shamir Medical Center, Tel Aviv, Israel

**Keywords:** PBMC microRNA expression, peripheral blood mononuclear cells, endurance athletes, runners, cyclists, females

**Affiliation** Department of Biomedical Engineering, Faculty of Engineering, Tel Aviv University, Tel Aviv, Israel was omitted for **Author** Rawan Ibrahim. This **Affiliation** has now been added for **Author** Rawan Ibrahim.

**Author** Rawan Ibrahim was erroneously assigned to **Affiliation** Pediatric Infectious Diseases Unit, The Center for Microbiome Research, Shamir Medical Center, Tel Aviv, Israel. This **Affiliation** has now been removed for **Author** Rawan Ibrahim.

The original article has been updated.

